# Hepatitis C (HCV) prevalence in citizens of the Métis Nation of Ontario

**DOI:** 10.1186/s12879-024-09171-w

**Published:** 2024-03-06

**Authors:** Noel Tsui, Gabriel B. Tjong, Abigail J. Simms, Sarah A. Edwards, Shelley Cripps

**Affiliations:** 1 Métis Nation of Ontario, 66 Slater Street, Ottawa, ON K1P 5H1 Canada; 2 Dalla Lana School of Public Health, University of Toronto, 155 College St, Toronto, ON M5T 3M7 Canada; 3 ICES Central, V1 06, 2075 Bayview Avenue, Toronto, ON M4N 3M5 Canada

**Keywords:** Hepatitis C, Métis, Indigenous health, HCV infection, Population-based

## Abstract

**Background:**

Hepatitis C virus (HCV) infection is a major global concern, with Indigenous Peoples bearing the highest burden. Previous studies exploring HCV prevalence within Indigenous populations have predominantly used a pan-Indigenous approach, consequently resulting in limited availability of Métis-specific HCV data. The Métis are one of the three recognized groups of Indigenous Peoples in Canada with a distinct history and language. The Métis Nation of Ontario (MNO) is the only recognized Métis government in Ontario. This study aims to examine the prevalence of self-reported HCV testing and positive results among citizens of the MNO, as well as to explore the association between sociodemographic variables and HCV testing and positive results.

**Methods:**

A population-based online survey was implemented by the MNO using their citizenship registry between May 6 and June 13, 2022. The survey included questions about hepatitis C testing and results, socio-demographics, and other health related outcomes. Census sampling was used, and 3,206 MNO citizens responded to the hepatitis C-related questions. Descriptive statistics and bivariate analysis were used to analyze the survey data.

**Results:**

Among the respondents, 827 (25.8%, CI: 24.3–27.3) reported having undergone HCV testing and 58 indicated testing positive, resulting in a prevalence of 1.8% (CI: 1.3–2.3). Respondents with a strong sense of community belonging, higher education levels, and lower household income were more likely to report having undergone HCV testing. Among those who had undergone testing, older age groups, individuals with lower education levels, and retired individuals were more likely to test positive for HCV.

**Conclusion:**

This study is the first Métis-led and Métis-specific study to report on HCV prevalence among Métis citizens. This research contributes to the knowledge base for Métis health and will support the MNO’s health promotion program and resources for HCV. Future research will examine the actual HCV incidence and prevalence among MNO citizens.

## Background

Hepatitis C virus (HCV) is considered one of the most burdensome infections in Canada, with an estimated 388,000 individuals (1% of the population) infected with HCV nationally [1] and 36.5 cases of HCV per 100,000 people in Ontario [2]. Among this 1% of the population, Indigenous Peoples (First Nation, Inuit, and Métis) accounted for 7%, with 1 in 30 Indigenous people living with chronic hepatitis C [1]. There is a paucity of literature on the epidemiology of hepatitis C in Indigenous Peoples in Canada, and of those that have been conducted most focus on First Nations populations [3–7]. Additionally, a systematic review conducted byBruce et al. [8] found that HCV infection rates varied widely in American Indian and Alaska Native populations in the United States and Indigenous populations in Canada. This variation is linked to the sample population of the studies, where some specifically targeted people who inject drugs (PWID), leading to higher HCV prevalence, while others were conducted with the general Indigenous population, resulting in a lower HCV prevalence [8]. Adding to this finding,Shaw et al. [9] found that among Indigenous people in Canada who inject drugs, a higher percent had HCV infection if they also used solvents. These studies help narrow down which populations among Indigenous people in Canada are most likely to be impacted by HCV infection. However, they both use pan-Indigenous samples, so the distinct stories of First Nations, Inuit, and Métis peoples cannot be distilled from this data.

The Métis are a post-contact Indigenous Nation that emerged from relations between First Nations women and European men. Historic communities formed from Ontario westward to British Columbia and to parts of the Northwest Territories and the Northern United States. The Métis have a unique history, language, and culture, and are one of the three recognized groups of Indigenous Peoples in Canada [10, 11]. Ontario is one of the ten provinces in Canada, located in east-central Canada. The Métis Nation of Ontario (MNO), established in 1993, is the only recognized Métis government in Ontario. There are also the Manitoba Métis Federation, the Métis Nation Saskatchewan, the Métis Nation of Alberta, and the Métis Nation British Columbia serving Métis citizens across Canada. The national definition of Métis, which is used by the MNO to determine citizenship, is (1) self-identifies as Métis, (2) is distinct from other Aboriginal peoples, (3) is of Historic Métis Nation ancestry, (4) is accepted by the Métis Nation. The MNO represents the collective aspirations, rights, and interests of over 30,000 Métis citizens [11]. The MNO provides a number of social, justice, and educational services to their citizens and conducts research on their behalf.

It is well understood that there is a lack of Métis-specific health research [12–14]. Moreover, when it comes to communicable diseases such as sexually transmitted and blood-borne infections (STBBI), including hepatitis C, there is a paucity of available data at both the provincial (Ontario) and national levels regarding the incidence and prevalence for Métis populations. Recognizing the scarcity of Métis-specific data on hepatitis C, the MNO included a self-reported HCV testing and results question on a population-based survey of MNO citizens in 2022. This study aims to examine the prevalence of self-reported HCV testing and positive results among MNO citizens. It is imperative for the MNO to continue collecting and enhancing hepatitis C data to inform prevention programs and provide supports for affected MNO citizens.

## Methods

### Study design and setting

This study was conducted as part of an MNO-led, population-based online survey which began May 6th and closed June 13th, 2022. The MNO maintains the only recognized provincial registry for Métis people in Ontario. At the time of the survey, there were 27,780 registered MNO citizens. Census sampling was used to invite all MNO citizens aged 16 and older with a valid email on file (*N* = 15,214) to complete the survey, which was deployed on Qualtrics. Prior to receiving the email invitation, all eligible MNO citizens with a valid phone number were notified about the survey through an automated phone call. The survey was also advertised through the MNO website and various social media platforms such as Twitter, Facebook, and Instagram. All respondents received a $5 coffee gift card upon completion of the survey and were entered into a draw for a chance to win one of 50 $100 Visa gift cards.

The survey underwent thorough review by MNO leadership and staff members, three MNO Senators, and obtained approval from the Provisional Council of the MNO Executive (PCMNO). The study adhered to the ethical principles outlined for Métis research [15] and received ethical approval from the University of Toronto Health Sciences Research Ethics Board (#42,320).

## Measures

The primary outcome was MNO citizens’ self-reported hepatitis C testing and testing results. Respondents were asked two questions: “Have you ever been tested for Hepatitis C viral infection?” and “Have you ever tested positive for Hepatitis C?” (only available to respondents that answered ‘yes’ to the previous question). Response options included yes, no, not sure, prefer not to say, and the option to skip the question.

In addition to the hepatitis C-related questions, sociodemographic information including gender, education level, employment status, household income, and relationship status were collected. Age was linked from the MNO Registry. Furthermore, the survey included a question related to Métis culture, specifically asking respondents to rate their sense of belonging to Métis community. Response options for this question included very strong, somewhat strong, somewhat weak, very weak, not sure, and prefer not to say.

### Statistical analysis

Descriptive statistics were used to describe the sample of MNO citizens. Chi-square and Fisher’s exact test were used to explore differences in testing and testing positive by sociodemographic variables. Data analysis was performed using SAS 9.4.

## Results

### Respondents

During the study period, there were 15,214 (55%) MNO citizens with a valid email on file. Within that, 4,780 (31%) MNO citizens followed the survey link, 4,209 (28%) completed the entire survey, and 76% (3206/4209) answered the hepatitis C-related questions.

### Respondent characteristics

The demographics of the hepatitis C questions respondents are presented in Table 1. More than half of the respondents were women (56.4%), were between the ages of 30 and 64 (66.6%), were in a relationship (72.0%), had full-time employment or were self-employed (50.1%), and held a college or university or professional degree (59.0%). Additionally, almost half of the respondents expressed a strong sense of belonging to Métis community (40.4%) and reported a household income between $50,000 to $99,999 (48.9%).

**Table 1:** Demographic characteristic of respondent to the Hepatitis C infection question (*N* = 3206) from survey

Characteristic	HCV Testing - Respondent (*N* = 3206)		p-value	Survey - Respondent (*N* = 4209)
	No (2379)	Yes (827)		
**Age**			0.0004	
16–29	341 (76.29)	106 (23.71)		588 (15.19)
30–49	824 (70.49)	345 (29.51)		1480 (38.23)
50–64	719 (74.43)	247 (25.57)		1108 (28.62)
65+	495 (79.33)	129 (20.67)		695 (17.95)
**Relationship**			0.0872	
Single	310 (71.59)	123 (28.41)		524 (14.17)
Widowed or divorced	199 (69.82)	86 (30.18)		347 (9.39)
In a relationship	1729 (74.91)	579 (25.09)		2826 (76.44)
**Income**			0.0048	
$49,999 or less	214 (65.44)	113 (34.56)		1080 (33.4)
$50,000 - $99,999	1150 (73.34)	418 (26.66)		1203 (37.2)
$100,000 or more	570 (74.9)	191 (25.1)		951 (29.41)
**Gender**			0.1836	
Man	862 (73.99)	303 (26.01)		1412 (38.1)
Women	1344 (74.34)	464 (25.66)		2221 (59.93)
2-Spirit/ Non-Binary	41 (64.06)	23 (35.94)		73 (1.97)
**Employment**		< 0.0001		
Student	81 (80.2)	20 (19.8)		125 (3.37)
Retired	549 (79.91)	138 (20.09)		817 (22.0)
Full time or self-employed	1183 (73.62)	424 (26.38)		1976 (53.22)
Part-time	146 (70.19)	62 (29.81)		256 (6.89)
Homemaker, full time parent or other	232 (65.17)	124 (34.83)		437 (11.77)
Unemployed	52 (70.27)	22 (29.73)		102 (2.75)
**Education**			< 0.0001	
High school or less	702 (79.23)	184 (20.77)		1083 (29.24)
Red seal / trades	181 (72.69)	68 (27.31)		293 (7.91)
College or university	1104 (70.81)	455 (29.19)		1913 (51.65)
Professional	249 (75.0)	83 (25.0)		415 (11.2)
**Sense of belonging**		0.0412		
Strong	948 (73.26)	346 (26.74)		1570 (42.24)
Weak	1081 (73.34)	393 (26.66)		1808 (48.64)
Not sure	217 (80.37)	53 (19.63)		339 (9.12)

### Hepatitis C testing and results

Among those who responded to the HCV questions, approximately 1 in 4 (25.8%, 95% CI: 24.3–27.3) indicated that they had been tested for Hepatitis C viral infection, with 58 reporting a positive test result, resulting in a prevalence of 1.8% (95% CI: 1.3–2.3). Those who had undergone HCV testing were more likely to have a strong sense of community belonging (*p* = 0.0412), a higher level of education (*p* < 0.0001), and a lower household income (*p* = 0.0048). In contrast, respondents aged 16 to 29 and those aged 65 and above (*p* = 0.0004), who were students or retired (*p* < 0.0001) during data collection, were less likely to have undergone HCV testing.

Among respondents who had undergone HCV testing (demographics are presented in Table 2), there was a higher likelihood of older age groups (*p* = 0.0002), individuals with a lower level of education (*p* = 0.0225), and retired individuals (*p* = 0.0030) to have tested positive for HCV. Given the small sample size, demographic characteristics such as age, gender, education, and sense of belonging have been grouped together to mitigate the risk of re-identification.

**Table 2:** Demographic characteristics of respondents to the HCV testing question (*N* = 827) from survey

Characteristic	Positive HCV Test Result		p-value
	No (769)	Yes (58)	
**Age**			0.0002
16–49	434 (96%)	17 (4%)	
50–64	223 (90%)	24 (10%)	
65+	112 (87%)	17 (13%)	
**Relationship**			0.8933
Single	115 (93%)	8 (7%)	
Widowed or divorced	79 (92%)	7 (8%)	
In a relationship	539 (93%)	40 (7%)	
**Income**			0.1586
$49,999 or less	100 (89%)	13 (11%)	
$50,000 - $99,999	389 (93%)	29 (7%)	
$100,000 or more	180 (94%)	11 (6%)	
**Gender**			0.2616
Man	278 (92%)	25 (8%)	
Women or Non-Binary	457 (94%)	30 (6%)	
**Employment**			0.0030
Student	20 (100%)	0 (0)	
Retired	121 (88%)	17 (12%)	
Full time or self-employed	406 (96%)	18 (4%)	
Part-time or unemployed	77 (92%)	7 (8%)	
Homemaker, full time parent or other	110 (89%)	14 (11%)	
**Education**			0.0225
High school or less	164 (89%)	20 (11%)	
Post-secondary or higher	570 (94%)	36 (6%)	
**Sense of belonging**			0.6823
Strong	323 (93%)	23 (7%)	
Weak or not sure	413 (93%)	33 (7%)	

## Discussion

This is the first Métis-led and Métis-specific study examining the prevalence of HCV and the associations between sociodemographic variables and the likelihood of undergoing HCV testing and testing positive for HCV among MNO citizens. Overall, approximately 25% of respondents self-reported having been tested for HCV and 58 individuals indicated a positive test result, resulting in a prevalence of 1.8%.

Our findings reveal that respondents who had a strong sense of community belonging, higher educational attainment, and lower household income were more likely to report having undergone HCV testing. This suggests that community engagement, educational opportunities, and financial resources for lower-income families may contribute to promoting health-seeking behaviours and enhancing access to HCV testing for MNO citizens.

In addition, our study indicates that respondents in older age groups (aged 50 and above), with lower levels of education, and retired individuals were more likely to report testing positive for HCV. This finding aligns with studies conducted on the general population in Ontario, where an increased prevalence of HCV has been observed among individuals aged 60–69 [2] with individuals born between 1950 and 1964 having a higher likelihood of testing positive for HCV [16]. Furthermore, it is important to note that injection drug use accounts for 60–85% of new HCV infections in Canada [17–19]. Lourenço et al. [18] has also shown that injection drug use is associated with lower levels of education and lower income. Furthermore, Graham et al. [20] has shown that the highest prevalence estimates of anti-HCV were among Australian Aboriginal and Torres Strait Islanders who inject drugs. However, this study did not collect data on injection drug use or other routes of HCV transmission, and further exploration of this relationship for MNO citizens will be considered in future research.

Nonetheless, these results contrast with previous studies conducted with First Nations communities where HCV infections were more commonly observed in younger age groups and had a higher prevalence among women [3, 7, 21]. These discrepancies highlight the complexity and variability of HCV infection dynamics within different populations, including Indigenous Peoples, and emphasize the need for tailored health promotion and intervention strategies.

A major strength of this study is that it is the first to look at hepatitis C prevalence in Métis with a population-based approach. In addition, this study was led by the MNO; the MNO was involved throughout the entire research process, which ensured adherence to ethical principles of Métis research and as a result supports Indigenous data sovereignty and the rights of Indigenous Peoples to have control over various aspects of data, including collection, storage, ownership, access and consents, and application [22].

## Limitations

The respondents only represent a sample of MNO citizens, which may have excluded individuals with HCV. Furthermore, the survey did not collect information on the respondents’ location (urban vs. rural/remote areas), which may have contributed to the low testing rate and prevalence due to limited technology access and limited access to HCV testing opportunities and resources. Another limitation is the reliance on self-reported hepatitis C testing and results, both of which were optional to complete, which may have resulted in response bias. The stigma associated with HCV, as well as its connection to HIV and the perception of patients as underserving or unworthy of help, could have influenced respondents’ willingness to disclose accurate information [23, 24]. This study also did not collect data on injectable drug use, which may have resulted in an underestimation of the calculated prevalence of HCV. Generally, PWID are more likely to have undergone HCV testing and have positive results [25], and those results may not have been captured in this study. Furthermore, the unawareness of HCV status among PWID [26, 27] might further contribute to the underestimated prevalence. Last, although the survey captured a broad array of socio-demographics, including community belonging, it did not ask questions probing all Métis social determinants of health which may be important for understanding HCV testing and results. Future research led by the MNO will examine HCV incidence and prevalence among MNO citizens with data linkage between the MNO citizenship registry and Ontario reportable disease data held within ICES.

## Conclusion

This is the first Métis-specific study to describe the prevalence of HCV testing and positive HCV results among MNO citizens. Our findings indicate that 25% of respondents have undergone HCV testing, with 58 testing positive, resulting in a prevalence of 1.8%. These findings highlight the importance of community belonging, educational attainment, income levels, age, and employment status in influencing individuals’ likelihood of undergoing HCV testing and testing positive. These results offer valuable insights and contribute to bridging the existing gaps in Métis health research. Future research will use data linkage to examine the full population of MNO citizens with respect to testing, incidence, and prevalence of hepatitis C, as well as collect stories and lived experiences from MNO citizens using *keeyoukaywin* – the visiting way methodology [28] which will inform health promotion, education, and advocacy efforts by the MNO.

## Data Availability

Given the tension releasing open data would create for the Métis Nation of Ontario (MNO) as all governing members from the Métis Nation walk the path towards self-government and self-determination, the data will not be available to everyone and will remain in the control of the MNO. Researchers interested in supporting Métis health research in collaboration with the MNO may request access and get approval for use. Please contact the author, SC, to request access to the data from this study.
